# False HbA_1c_ value due to a rare variant of hemoglobin Petie Salpetriere coinherited with alpha thalassemia

**DOI:** 10.1515/almed-2024-0037

**Published:** 2024-10-09

**Authors:** Esperanza Lepe Balsalobre, Gema María Varo Sánchez, Marta Rico Rodríguez, Sandra Fuentes Cantero

**Affiliations:** Department of Laboratory Service, Área de Gestión Sanitaria Norte de Huelva, Hospital de Riotinto, Minas de Riotinto, Huelva, Spain

**Keywords:** variant, hemoglobin, interference, glucose, diabetes

## Abstract

**Objectives:**

To describe a variant hemoglobin that interferes with HbA_1c_ analysis by cation exchange HPLC.

**Case presentation:**

A 78 years-old Spanish male patient visited the Internal Medicine Clinic for a routine check-up, with HbA_1c_ included to screen for diabetes. He had suffered hypertension and dyslipidemia, and the patient had no previous symptoms suggestive of diabetes such as hyperglycemia, weight loss, polydipsia, polyuria or tiredness. Diabetes screening by HbA_1c_ measurement was assessed using cation exchange HPLC and an immunoassay point-of-care analyzer. Routine hemoglobinopathy screening was performed including CBC, HbF and HbA_2_ measurement by cation exchange HPLC and capillary electrophoresis (CE). Further variant characterization was undertaken by DNA sequencing. Discordant HbA_1c_ results were obtained for our subject, with elevated HbA_1c_ of 52 mmol/mol measured by cation exchange HPLC and a normal level of 34 mmol/mol by immunoassay. Abnormal HbA_1c_ peak shape prompted hemoglobinopathy screening to investigate potential variant interference. A globin gene analysis was performed, and the results showed a variant hemoglobin named ‘Hb Petie Salpetriere’. This variant arises from a Val → Phe substitution due to a mutation of c.103G>T of the beta-globin gene [BETA34 (B16) Val>Phe; HBB:c.103G>T].

**Conclusions:**

This is the first reported case involving the Hb Petie Salpetriere variant in a Spanish patient. The present results show that the Hb Petie Salpetriere variant can affect the results of HbA_1c_ analysis through ion-exchange HPLC, but not that obtained from the latex agglutination immunoassay. Only ion-exchange HPLC suggested the presence of the Hb variant in this case, suggesting that a careful review of the resulting chromatogram might reveal a potential variant.

## Introduction

Glycated hemoglobin (HbA_1c_) is the most used analyte to monitor glycemic control in diabetic patients. It is also used for diagnosis of diabetes. The accuracy of some methods may be affected by the presence of hemoglobinopathies. Term hemoglobinopathy includes all hemoglobin (Hb) genetic disorders: thalassemic and abnormal hemoglobins. It is estimated that in the year 2000 there were 171 million individuals with diabetes mellitus in the world, in 2030 this figure will reach 366 million. Approximately 7 % of the world population are heterozygous carriers of hemoglobin disorders. The most common variants in the world in descending order of prevalence are HbS, HbE, HbC and HbD. Some hemoglobin variants are associated with disease, although the majority are clinically silent and are discovered by chance, sometimes when measuring HbA_1c_. In cases of heterozygosity, the survival of the erythrocytes is usually normal, so the HbA_1c_ measurement is adequate for glycemic control, as long as the variant does not interfere with the method used or with the union of glucose to hemoglobin [1, 2].

## Case presentation

A 78 years-old Spanish male patient visited the Internal Medicine Clinic for a routine check-up, with HbA_1c_ included to screen for diabetes. He had suffered hypertension and dyslipidemia, and the patient had no previous symptoms suggestive of diabetes such as hyperglycemia, weight loss, polydipsia, polyuria or tiredness.

**Figure 1:**
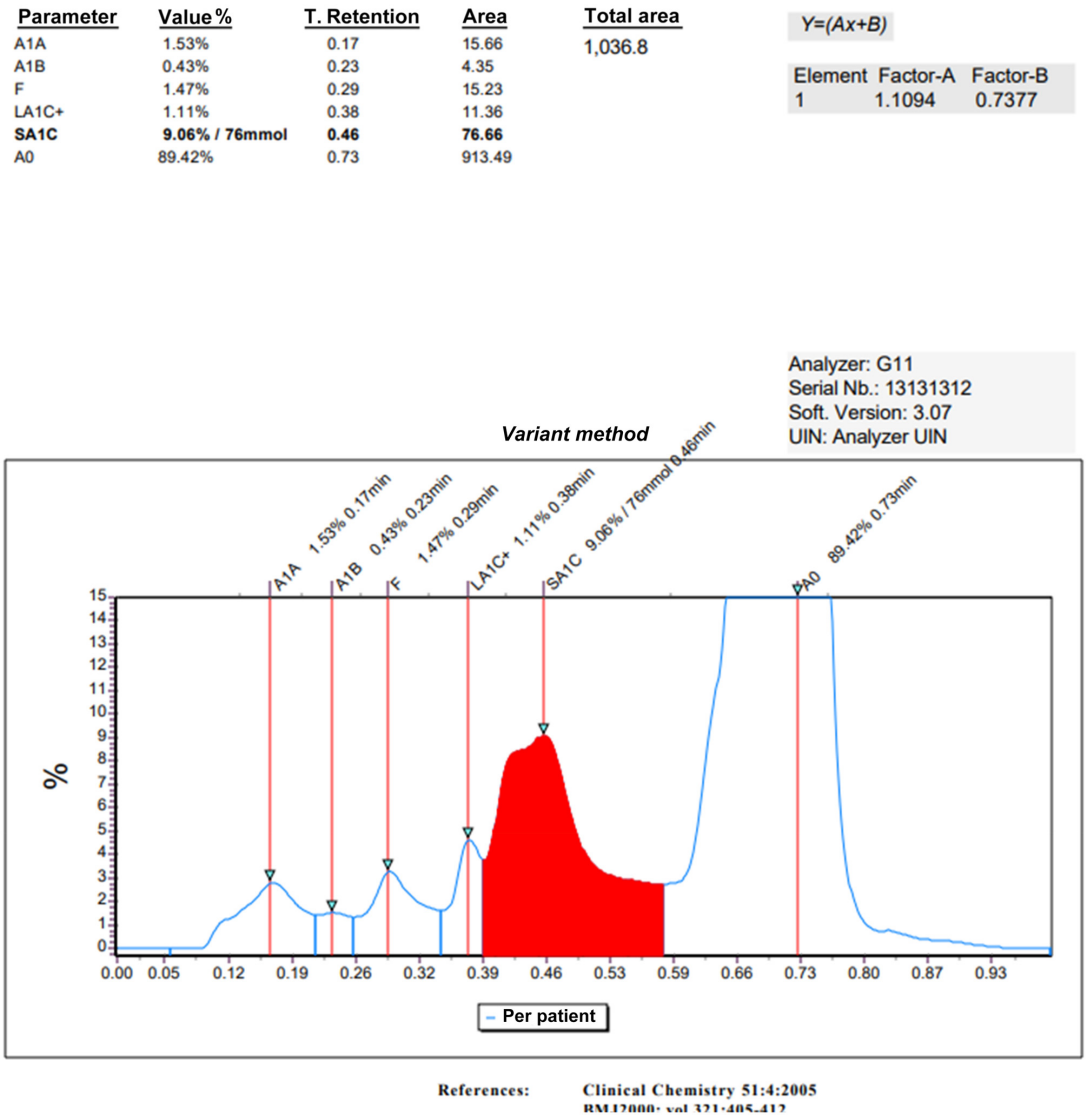
Chromatogram with the Hb variant detected.

The HbA_1c_ value was 77 mmol/mol (9.1 %) measured in our ion exchange high-performance liquid chromatography (HPLC) analyser (Tosoh G11, Horiba, Japan) (Figure 1). His fasting blood glucose was 5.77 mmol/L (upper limit reference interval, 6.1 mmol/L) and haemoglobin electrophoresis unremarkable. The remaining blood tests showed: an haemoglobin of 181 g/L, a hematocrit of 0.549 L/L, a slight macrocytosis (due to vitamin B12 (cobalamin) deficiency) (medium corpuscular volumen of 101.7 fL, normal range: 82–96 fL) with hyperchromia (medium corpuscular haemoglobin of 33.5 pg, normal range: 27–31 pg).

The HbA_1c_ measured by latex agglutination immunoassay (Cobas b101, Roche Diagnostics, Switzerland) was 25 mmol/mol (4.3 %), which was more consistent with his serial blood glucose values. Non other biochemical parameters showed significant alterations.

Since the abnormal chromatography indicated a suspected hemoglobin variant, blood samples were sent to a thalassemia and hemoglobinopathy reference laboratory for molecular characterization. Globin chain were studied by reverse-phase HPLC and the study revealed a loss of an alpha gene in an allele due to deletion of 3.7 KB (-ALPHA3.7/ALPHA ALPHA). Besides, a globin gene analysis was performed, and the results showed a variant hemoglobin named ‘Hb Petie Salpetriere’. This variant arises from a Val → Phe substitution due to a mutation of c.103G>T of the beta-globin gene [BETA34 (B16) Val>Phe; HBB:c.103G>T]. In addition, there was a chance discovery of a heterocytic alpha-associated thalassemia.

## Discussion

The suspicion of the presence of a hemoglobinopathy in the patient during the HbA_1c_ analysis led to the definitive identification of a structural variant of the beta globin chain, known as ‘Hb Petie Salpetriere’. Consulting the Database of Human Hemoglobin Variants and Thalassemia (HbVar) shows that only two cases of this variant have been described in the literature: one case in Japan and another in France. Therefore, we are facing the first described case of this variant in our country [3, 4].

Hb Petie Salpetriere is substituted at position 34 of the beta chain, which is directly involved in the ɑ1ß1 contact. A substitution in this region causes an allosteric imbalance of hemoglobin, which causes the loss of bonds in these contacts, loosening the deoxy quaternary structure. The type of amino acid substitution, its position, and the proportion of mutant hemoglobin influence the effect produced on the functionality of the molecule because residue 34 is involved in the binding of the β globin chain with heme. Specifically, Hb Petie Salpetriere has a high affinity for oxygen (10-fold increased), a decreased cooperativity, a decreased Bohr effect and a normal or slightly decreased 2,3-diphosphoglycerate effect. Generally, high affinity variants become fully saturated with oxygen in the lung, but at a tissue capillary pO2 of 35–45 mm Hg, they deliver less oxygen than normal Hb A. This results in mild tissue hypoxia which stimulates increased erythropoietin production and subsequent polycythemia [5, 6].

Haemoglobinopathies with altered oxygen affinity are rare diseases, but can easily be suspected from the findings of simple laboratory investigations such as the measurement of p50. In our opinion, this parameter should be routinely investigated in patients with erythrocytosis, especially in young patients in whom polycythemia vera is considered statistically unlikely, or at least after it has been excluded by clinical and laboratory criteria 5], [6], [7.

## Lessons learned

In conclusion, this is the first reported case involving the Hb Petie Salpetriere variant in a Spanish patient. The present results show that the Hb Petie Salpetriere variant can affect the results of HbA_1c_ analysis through ion-exchange HPLC, but not that obtained from the latex agglutination immunoassay. The inclusion of plasma glucose level can assist with interpretation of the HbA_1c_ values. Furthermore, only ion-exchange HPLC suggested the presence of the Hb variant in this case, suggesting that a careful review of the resulting chromatogram might reveal a potential variant. Finally, a genetic evaluation can confirm an Hb variant.
